# Why does the world continue to struggle with effectively addressing hepatitis E?

**DOI:** 10.17179/excli2025-9166

**Published:** 2026-01-08

**Authors:** Henrique Borges da Silva Grisard, Gislaine Fongaro

**Affiliations:** 1 Federal University of Santa Catarina, Santa Catarina State, 88052162, Brazil

## ⁯⁯

Hepatitis E virus (HEV) remains a globally neglected public health concern, causing up to 70,000 deaths each year (CDC, 2024[2]). Its clinical presentation ranges from asymptomatic infection to acute liver failure, chronic hepatitis, and fatal outcomes, particularly in immunocompromised individuals and pregnant women; while the disease is often self-limiting in immunocompetent adults, prognosis worsens substantially in these high-risk groups. A central problem and the main reason HEV remain largely invisible in public health agendas is the worldwide difficulty in diagnosing the infection. Across regions, a striking lack of sensitive, specific, and standardized diagnostic assays exists, resulting in inconsistent results, underreporting, and unreliable epidemiological data. Consequently, clinicians often do not consider HEV as a differential diagnosis, perpetuating a cycle in which the virus remains undetected, and its true burden is underestimated (Buchardt et al., 2025[1]; Damaris et al., 2022[3]; Kar and Karna, 2020[4]; Wu et al., 2020[7]).

A significant barrier in HEV diagnosis stems from the shortfall of approved, sensitive, and specific assays worldwide, which leads to diagnostic inconsistencies and the underdiagnosis of HEV infections. This results in a vicious cycle where HEV is not detected, leading to a lack of epidemiological data, which in turn prevents health professionals from suspecting HEV as the causative agent of such diseases. Even though nucleic acid amplification tests, such as Reverse Transcribed Real-Time Polymerase Chain Reaction (RT-qPCR), for detecting HEV's genome is the most specific way to confirm its infection, the requirement of trained personnel and specialized equipment, as well as its high cost, limits usefulness of this method. With that, a reliance on serological tests (anti-HEV IgM and IgG) is necessitated, which is suboptimal since these tests exhibit poor performance and stability (Kar and Karna, 2020[4]).

In immunocompromised patients, including people living with HIV and organ transplant recipients, there is a high risk of chronic HEV infection, which can lead to cirrhosis and hepatocellular carcinoma. An often-used treatment for this group is ribavirin therapy alongside supportive care, but increases in resistance and treatment failure undermine the potential of such therapy. Pregnant women face exceptionally detrimental outcomes from HEV; in developing countries, HEV infections during pregnancy can lead to fatality rates from 20 % to 30 %, particularly in the third trimester (Lhomme et al., 2016[5]). Complications can include acute liver failure, eclampsia, hemorrhage, and pre-/perinatal complications such as preterm birth and stillbirth (Wen et al., 2023[6]; Wu et al., 2020[7]). Unfortunately, ribavirin can't be used given its contraindication for this population, so the primary form of treatment is supportive care, relying solely on the person's immune system.

Although an effective vaccine (Hecolin®) is available in China, its limited global dissemination restricts its public health impact, reinforcing the need for expanded research and implementation strategies, particularly in highly vulnerable regions. Therefore, we urge policymakers, healthcare professionals, and researchers worldwide to prioritize investments in standardized diagnostic tools, coordinated One Health surveillance strategies, targeted interventions in epidemiological hotspots, and improved therapeutic options, particularly for high-risk groups. Such collective action is essential to ensure timely interventions, enhance epidemiological accuracy, and more effectively protect vulnerable populations from the substantial and still underrecognized burden of HEV.

Achieving the World Health Organization's goal of eliminating viral hepatitis by 2030 will not be possible without a coordinated global effort to strengthen HEV detection, treatment, and surveillance, including the prioritization of hotspots with high transmission and disease. Advancing this agenda also requires a truly integrated approach that incorporates human, animal, and environmental diagnostic strategies, given the well-recognized zoonotic dynamics and environmental circulation of the virus factors that remain largely overlooked. Taken together, these considerations underscore the urgent need for rapid, accurate, and accessible HEV diagnostic methods, as well as the development of effective and safe antiviral options.

## Declaration

### Financial support

The scholarship for HBSG was funded by Fundação de Amparo à Pesquisa e Inovação do Estado de Santa Catarina (FAPESC).

### Acknowledgments

None.

### Conflict of interest

The authors declare no competing interests.

### Artificial Intelligence (AI) - assisted technology

The authors declare that no artificial intelligence tools were used in the preparation of this manuscript.

### Author contributions

Henrique Borges da Silva Grisard: original draft preparation. Gislaine Fongaro: conceptualization, supervision, review and editing. Both authors read and approved the final manuscript.
